# Management of non-medical androgen use: a position statement by the Society for Endocrinology

**DOI:** 10.1530/EC-26-0361

**Published:** 2026-09-22

**Authors:** Bonnie Grant, Channa N Jayasena, Satyajit Nag, Richard Quinton

**Affiliations:** ^1^ Section of Investigative Medicine, Department of Metabolism, Digestion and Reproduction, Imperial College London, London, United Kingdom; ^2^ James Cook University Hospital, South Tees Hospitals NHS Foundation Trust, Middlesbrough, United Kingdom

**Keywords:** androgen abuse, anabolic–androgenic steroids, testosterone, hypogonadism, sexual dysfunction, depression, withdrawal

## Abstract

Non-medical androgen use, commonly involving anabolic–androgenic steroids, represents an increasing public health concern. Non-medical androgen use is associated with significant morbidity and mortality, including cardiovascular disease, erythrocytosis, dyslipidaemia, infertility, sexual dysfunction, psychiatric manifestations and suppression of the hypothalamic–pituitary–gonadal axis. Men with current or previous non-medical androgen use are increasingly presenting to endocrine and reproductive medicine services, often with hypogonadal symptoms, infertility or concerns regarding recovery following cessation. However, both patients and clinicians frequently report mistrust, uncertainty and limited confidence in management approaches. This Society for Endocrinology position statement summarises the currently available evidence relating to non-medical androgen use, associated health consequences, biochemical recovery following cessation and potential management strategies. Current evidence suggests that most men will experience biochemical recovery of the HPG axis within approximately 12 months of androgen cessation, although recovery timelines vary substantially and some individuals may develop persistent hypogonadism. Symptoms such as low mood, fatigue, anxiety and sexual dysfunction may persist despite biochemical recovery and are likely influenced by psychological factors in addition to serum testosterone concentrations. Existing evidence supporting pharmacological interventions, including human chorionic gonadotrophin, selective oestrogen receptor modulators, aromatase inhibitors or testosterone therapy, remains limited and of low quality, with no robust evidence demonstrating improved long-term recovery or symptom outcomes. Clinicians should approach individuals who misuse androgens with compassion, professionalism and a non-judgemental attitude while remaining alert to undisclosed androgen use. Further longitudinal and interventional studies are required to better characterise recovery trajectories, identify predictors of persistent morbidity and inform evidence-based management strategies.

## Background

Non-medical androgen use, defined as the non-prescribed use of androgens outside approved medical indications, commonly involves anabolic–androgenic steroids in combination with other image- and performance-enhancing drugs (IPEDs). It is a growing public health concern, with the lifetime prevalence of non-medical androgen use estimated at approximately 6% of men globally, with higher rates reported among individuals participating in recreational and strength-based sports (1). In the United Kingdom, it has been estimated that around 500,000 men have engaged in non-medical androgen use, although the true prevalence is likely to be higher due to under-reporting and non-disclosure associated with the illicit and stigmatising nature of use (2).

In the UK, androgens are classified as Class C controlled drugs under the Misuse of Drugs Act 1971 and are listed in Schedule 4 (Part II) of the Misuse of Drugs Regulations 2001. Personal possession for self-administration is not illegal; however, it is illegal to manufacture, sell or supply these substances without appropriate authorisation, offences that may carry a maximum penalty of up to 14 years’ imprisonment (3). The only androgenic drugs having a marketing authorisation in the UK are testosterone esters (for male hypogonadism) and nandrolone (for osteoporosis, although this indication has been entirely superseded) (4, 5).

Most individuals initiate non-medical androgen use in early adulthood, typically in their twenties, but there is increasing concern regarding use among adolescents and young adults (6, 7). Individuals initiate androgen misuse for a variety of reasons, including increasing muscle mass and strength, improving physical appearance, enhancing sporting performance and, increasingly, self-management of non-specific symptoms attributed to low testosterone (6, 8). Androgens are frequently obtained via the internet or through informal networks, including gym-based peer groups (9). Identified risk factors for non-medical androgen use include body dysmorphia, childhood or adolescent conduct disorders, poor parental relationships and insecurities about sexual orientation (10, 11, 12, 13). Patterns of androgen use vary considerably, ranging from intermittent supraphysiological cycles to continuous (‘blast-and-cruise’) regimens (6).

Non-medical androgen use is associated with substantial morbidity and increased mortality. Observational studies suggest a three-fold increase in all-cause mortality among individuals with a history of non-medical androgen use, with cardiovascular disease representing the leading cause of death (14). Non-medical androgen use is also associated with erythrocytosis, dyslipidaemia, hypertension, cardiomyopathy, hepatotoxicity and, rarely, hepatic tumours, an increased risk of blood-borne viruses, psychiatric manifestations, infertility from azoospermia and oligospermia and sexual dysfunction (14, 15, 16, 17, 18, 19, 20, 21). Emerging evidence also suggests that prolonged androgen misuse may adversely affect brain health, with neuroimaging studies demonstrating structural brain changes, accelerated brain ageing and cognitive impairment in long-term users (22, 23).

Due to suppression of the hypothalamic–pituitary–gonadal (HPG) axis from supraphysiological androgen exposure, men with a history of non-medical androgen use are frequently referred to endocrine services, although the androgen use is not always signalled in the referral. Previous non-medical androgen use is an increasingly recognised cause of hypogonadism in clinical practice, with one andrology clinic reporting that 43% of men presenting with profound hypogonadism (total testosterone <1.7 nmol/L) reported prior non-medical androgen use (24). Indeed, the same biochemical profile can arise from ongoing use of non-testosterone androgens. Although patients often ascribe their troublesome symptoms to androgen deficiency, this may not always be the case, but for them to be able to consider an alternative explanation, the clinician must first establish a level of trust.

Individuals using androgens frequently report significant mistrust of healthcare professionals. It is also commonly perceived that even specialist clinicians lack the knowledge or expertise to manage complications related to non-medical androgen use and, indeed, many UK clinicians report limited training experience in reproductive endocrinology and may lack confidence in the assessment and management of this group (25, 26). Perceived stigma and previous negative experiences with healthcare professionals may discourage disclosure of androgen use and delay engagement with healthcare services (27). As a result, consultations can be fraught with uncertainty, tension and mistrust on both sides, which can act as a barrier to effective engagement and care.

Whilst remaining alert to clinical or laboratory indicators that are discrepant with the asserted medical history, clinicians should nevertheless aim to approach individuals who have abused androgens with compassion, professionalism and a non-judgemental attitude, delivering care that is consistent with the best currently available evidence. Acknowledging a patient’s concern regarding potential withdrawal symptoms and anticipating challenges during androgen cessation may improve engagement. The purpose of this position statement is to summarise currently available evidence relating to non-medical androgen use and its associated health consequences. At present, the evidence base remains insufficient to support the development of a formal clinical guideline; however, this statement seeks to provide a framework to inform clinical practice, support clinician education and identify future research priorities (Fig. 1).

**Figure 1:**
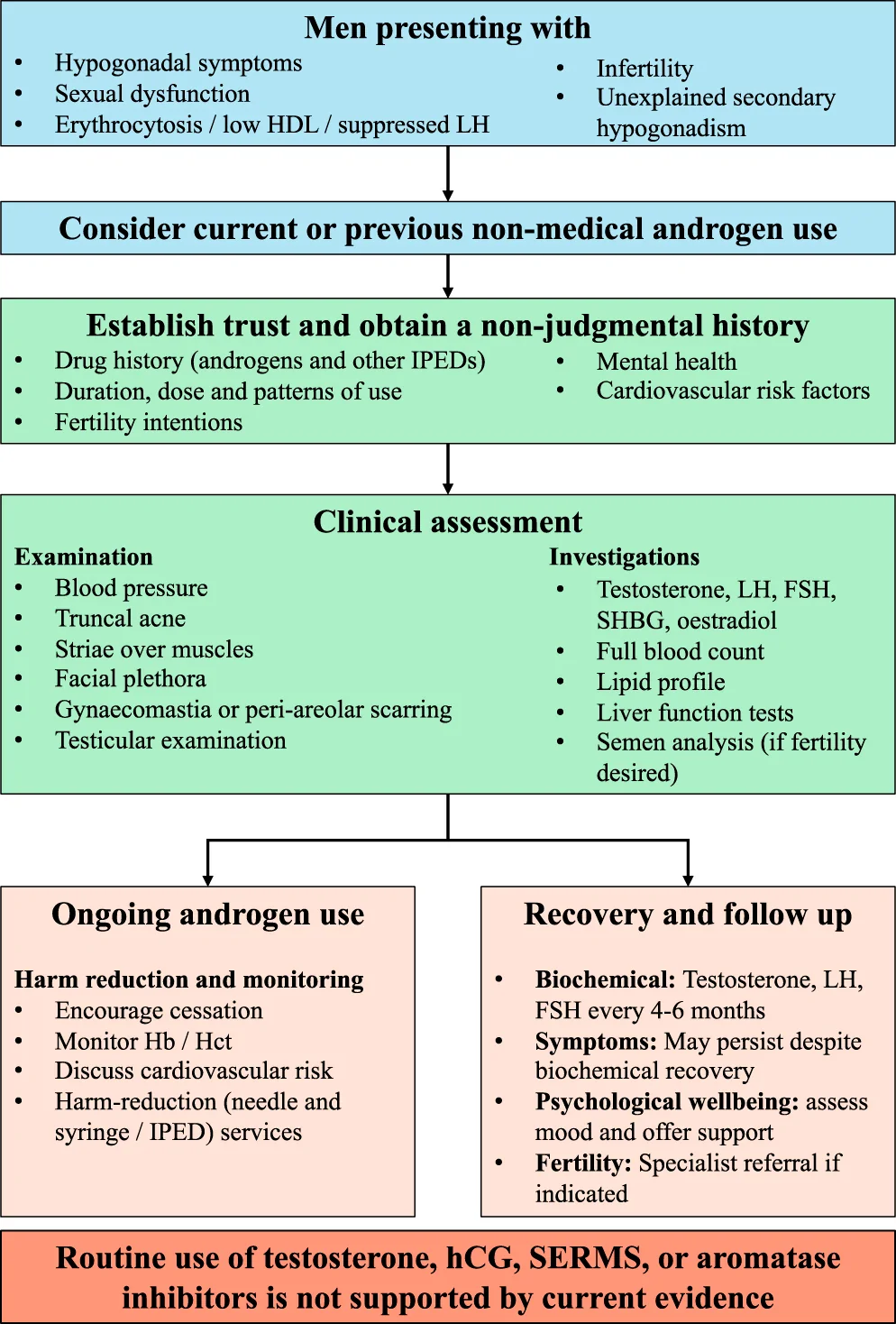
Suggested clinical approach to men with suspected current or previous non-medical androgen use. FSH, follicle-stimulating hormone; Hb, haemoglobin; hCG, human chorionic gonadotrophin; Hct, haematocrit; IPEDs, image- and performance-enhancing drugs; LH, luteinising hormone; SERMs, selective oestrogen receptor modulators; SHBG, sex hormone-binding globulin.

## Screening for undisclosed or inadvertent androgen use

Identification of undisclosed androgen use can be challenging in clinical practice. Shankara-Narayana *et al.* reported that self-reported non-use of androgens had a high negative predictive value (>80%), but in practice, under-reporting remains a concern in clinical settings, particularly where mistrust of healthcare professionals exists (28). A thorough clinical assessment is essential. Physical features that may raise suspicion of non-medical androgen use include truncal acne, disproportionate muscularity with prominent striae over large muscle groups (particularly the biceps), facial plethora and hypertension. Gynaecomastia may be present, and peri-areolar scarring may indicate previous surgical correction. Testicular volume is often preserved or only modestly reduced when non-medical androgen use began after puberty, although the consistency may be soft. Therefore, a markedly reduced testicular volume should prompt consideration and evaluation for an alternative cause of hypogonadism.

Certain laboratory features may also suggest current or recent androgen use. These include elevated haemoglobin and haematocrit, reduced sex hormone-binding globulin (SHBG), low HDL-cholesterol and elevated oestradiol concentrations from testosterone aromatisation. Abnormalities in liver function tests may raise suspicion of oral androgen use, which is more strongly associated with hepatotoxicity due to first-pass hepatic metabolism, but modest elevations in transaminases may also reflect recent intense exercise due to the release of muscle-specific alanine transaminase (ALT) (29). A markedly elevated serum testosterone concentration is strongly suggestive of exogenous androgen exposure, although serum testosterone levels may appear unexpectedly low if non-testosterone androgens (such as nandrolone) are being used exclusively, as these compounds are not detected by standard immunoassays. Shankara-Narayana *et al.* have reported that a single suppressed luteinising hormone (LH) level can correctly identify androgen use in approximately 89% of cases (28). Measurement of inhibin B, where available, may serve as a surrogate marker of Sertoli cell function. Semen analysis can be a useful tool, particularly in men seeking fertility, and may provide additional motivation for cessation by demonstrating the impact of non-medical androgen use on spermatogenesis. A urinary testosterone-to-epitestosterone (T/E) ratio, measured using mass spectrometry, is widely used in anti-doping to detect exogenous androgen use in professional sports but is not routinely available or validated in clinical practice (30). However, evidence for ongoing androgen use does not necessarily equate to lack of candour or disclosure by patients, as there may be inadvertent intake of undeclared androgens in dietary supplements targeted at this population of males (31).

For men with ongoing non-medical androgen use or recent cessation, safety monitoring is advisable. Haematological evaluation with haemoglobin and haematocrit measurements should be undertaken twice yearly. There is currently insufficient evidence to support routine therapeutic venesection for androgen-induced erythrocytosis and, indeed, this intervention may carry an increased thrombotic risk (32). Serial monitoring of serum testosterone and gonadotrophins at 4- to 6-month intervals may help demonstrate recovery of the HPG axis and may support ongoing patient engagement with care.

## Biochemical recovery following androgen cessation

The available evidence regarding biochemical recovery of the HPG axis following cessation of non-prescribed androgen use is limited and largely derived from observational and cross-sectional studies, with only one prospective study reported to date. A cross-sectional study by Shankara-Narayana *et al.* demonstrated a variable timeline for recovery of gonadotrophins and spermatogenesis following cessation of non-medical androgen use. In this cohort, the median recovery of LH occurred at approximately 10 months, with FSH recovery occurring by 12 months, although recovery of inhibin B concentrations and semen parameters was considerably slower, with some parameters taking up to 36 months to normalise (33). In contrast, Smit *et al.* reported that, in their cohort of men ceasing non-medical androgen use, the majority had biochemical parameters comparable to baseline within three months of cessation (34). Similarly, a scoping review by Solanki *et al.*, which included 36 studies, reported that gonadotrophin concentrations typically recover within 2–16 weeks following cessation of androgen exposure (35). In a large retrospective analysis of over 600 men, biochemical recovery following cessation of non-medical androgen use was more likely among individuals who had used fewer androgens, had a shorter duration of use or had ceased use for greater than 3 months (36).

Emerging evidence suggests that a subgroup of men may not achieve full biochemical recovery. Studies using human chorionic gonadotrophin (hCG) stimulation studies have demonstrated impaired testicular responsiveness in a small number of men (37, 38). The mechanisms underlying incomplete HPG axis recovery remain uncertain and may include direct testicular toxicity or indirect effects through cardiovascular or metabolic disease that are increased in men with a history of non-medical androgen use.

Taken together, the currently available evidence suggests that most men experience biochemical recovery of the HPG axis within approximately 12 months of androgen cessation (Table 1). However, recovery timelines vary substantially between individuals, and further longitudinal studies are required to better characterise the subgroup of men with persistent hypogonadism following non-medical androgen use.

**Table 1:** Estimated recovery timelines of laboratory parameters following cessation of non-medical androgen use.

Parameter	Recovery timeline	References
Total testosterone	3–30 months	34, 40, 41, 46
Luteinising hormone	3–13 months	33, 34, 45
Follicle-stimulating hormone	9–20 months	33, 34, 45
Sex hormone-binding globulin	9 months	34
Inhibin B	32 months	33
Sperm concentration	9–11 months	33, 34
Sperm motility	9–38 months	33, 34

## Withdrawal symptoms following androgen cessation

Symptoms including low mood, anxiety, sleep disturbances, fatigue and sexual dysfunction are commonly reported following cessation of non-medical androgen use (39). However, the timeline and trajectory of symptomatic recovery remain less clearly defined than those of biochemical recovery. Current evidence suggests that a substantial proportion of men report persistent symptoms despite discontinuation of androgens, in some cases for many years, even after biochemical recovery (40, 41). Men should be counselled that the course of recovery is highly variable and symptoms may persist for prolonged periods in some individuals. These symptoms are frequently cited as reasons for relapse of non-medical androgen use, with an estimated 30% of men going on to develop an androgen dependency syndrome (42). Scarth *et al.* have recently argued that androgen use disorder should be recognised as a distinct substance use disorder, reflecting its unique endocrine, behavioural and psychosocial characteristics, although this has not yet been incorporated into current diagnostic classification systems (43). Withdrawal symptoms may also adversely affect intimate relationships and contribute to partner or family distress, highlighting the importance of involving partners or family members in the recovery counselling pathway where appropriate (44).

The association between symptom severity and serum testosterone concentrations remains uncertain, and this should be conveyed to patients once trust has been established. In a large cross-sectional study, men with a history of non-medical androgen use within the preceding year reported higher levels of depression and anxiety, greater sexual dysfunction and poorer quality of life compared with non-using populations (45). Multivariable analysis demonstrated that the presence of a self-reported psychiatric comorbidity was the strongest predictor of depression, anxiety and reduced quality of life in this cohort, whereas serum testosterone concentrations showed only a modest association with depressive symptoms (45). Similarly, other studies have reported persistence of symptoms in some men despite serum total testosterone concentrations within the reference range (46). In contrast, a single cross-sectional study of men with a longer median time since cessation of non-medical androgen use (approximately two years) reported that higher age, lower serum total testosterone and shorter time since androgen cessation were all negatively associated with quality-of-life outcomes (41). In individuals presenting with severe depressive symptoms or suicidal ideation following androgen cessation, urgent mental health assessment and appropriate psychiatric input are required. While hypogonadism may contribute to psychological symptoms, observational studies from other hypogonadal populations suggest that psychological wellbeing is likely to reflect multiple disease- and patient-related factors rather than testosterone deficiency alone (47, 48). There is currently no evidence that testosterone therapy reduces suicide risk and therefore it should not be initiated solely for this indication.

These findings emphasise the importance of avoiding an overly simplistic attribution of symptoms solely to circulating testosterone concentrations, and both patients and clinicians may overestimate the direct relationship between testosterone levels and symptoms such as mood, wellbeing and sexual function in this cohort of men. Concurrent mental health conditions, expectations regarding androgen withdrawal gleaned from other users and the broader social and behavioural context of non-medical androgen use are likely to contribute substantially to persistent symptoms. However, the existing literature comprises largely retrospective studies with self-reporting, which limits causal inference. Longitudinal studies incorporating both biochemical markers and validated patient-reported outcome measures (PROMs) are required to better characterise the natural history of symptom recovery and identify individuals at risk of persistent morbidity.

## Non-gonadal recovery following androgen cessation

The main non-gonadal effects associated with non-medical androgen use are cardiovascular and metabolic. These include hypertension, dyslipidaemia (particularly reduced HDL-cholesterol), erythrocytosis and structural cardiac abnormalities, including cardiomyopathy (15). Available observational evidence suggests that many of these adverse effects improve and, in many cases, will fully reverse following sustained cessation of non-medical androgen use. Improvements in blood pressure, lipid and haematological parameters are commonly observed within 3–6 months of discontinuation (16). However, some individuals may experience persistence of some adverse effects, particularly structural cardiac abnormalities (33). The likelihood and extent of recovery may depend on several factors, including duration of exposure, pre-existing cardiovascular risk factors and comorbid disease. Clinicians should therefore consider cardiovascular risk assessment and appropriate follow-up in men with a history of prolonged or high-dose androgen exposure.

## Pharmacological interventions

There are currently no studies directly comparing gradual tapering versus abrupt cessation of non-prescribed androgen use. However, in practice, the pharmacokinetics of long-acting injectable preparations result in a gradual decline in circulating testosterone concentrations following cessation, effectively acting as a taper.

Many men who discontinue (or cycle in and out of) non-medical androgen use self-administer so-called ‘post-cycle therapy’ (PCT), typically consisting of human chorionic gonadotrophin (hCG), selective oestrogen receptor modulators (SERMs) or aromatase inhibitors (AIs) (39). These drugs are used to directly (hCG) or indirectly (SERMs and AIs) stimulate endogenous testosterone production, with the aim of accelerating HPG axis recovery and alleviating withdrawal symptoms. However, such approaches represent ongoing pharmacological manipulation of the HPG axis rather than true recovery, which is defined as restoration of endogenous function in the absence of treatment.

Rasmussen *et al.* randomised symptomatic men with a history of non-medical androgen use and low serum testosterone concentrations to letrozole (an aromatase inhibitor) or placebo (49). However, the study was terminated prematurely because of poor recruitment, with participants reportedly unwilling to accept the possibility of receiving placebo. Although letrozole increased serum testosterone concentrations during treatment, these improvements were not sustained following treatment withdrawal, and there were no clear improvements in sexual or depressive symptoms. However, the study was underpowered to determine clinical efficacy. Nevertheless, it highlights the considerable challenges of conducting placebo-controlled trials in this population. Overall, there remains a lack of adequately powered, randomised, double-blind studies evaluating the efficacy and safety of pharmacological agents in promoting sustained HPG axis recovery following cessation of non-medical androgen use.

Retrospective analyses have suggested higher short-term rates of biochemical recovery among men using hCG, SERMs and aromatase inhibitors; however, these findings are limited by study design and self-reporting by individuals and are confounded by the absence of toxicology testing, raising the possibility that observed effects reflect ongoing drug exposure rather than endogenous recovery (36). In a non-randomised, non-blinded study of 520 men treated with hCG, clomiphene citrate, tamoxifen and/or recombinant FSH, men in the treated group experienced improvements in serum testosterone and gonadotrophin levels (50). However, sustained recovery was incomplete, with only 32% of men responding to a single treatment course. Similarly, in studies evaluating hCG use during non-medical androgen use, spermatogenesis recovery was variable. Although 58% of men achieved a normal total motile sperm count at the end of treatment, the remainder remained oligo- or azoospermic (51).

Alternative approaches have been explored in men with severe withdrawal symptoms. One strategy involved temporary ‘bridging’ therapy using a combination of SERMs and hCG with short-term transdermal testosterone to mitigate withdrawal symptoms while also attempting to restore endogenous gonadotrophin secretion (52). Although LH and FSH concentrations increased into the reference range, only half of the participants achieved target testosterone concentrations by 16 weeks. Non-responders tended to be older and had a longer duration of androgen exposure. Notably, symptoms did not correlate with increases in serum testosterone concentrations on treatment (52).

Overall, there is currently a lack of robust evidence demonstrating that pharmacological interventions accelerate or sustain recovery of the HPG axis following cessation of non-medical androgen use. Furthermore, no well-designed study has shown that testosterone replacement therapy or alternative testosterone secretagogues lead to an improvement in symptoms in men with prior non-medical androgen use, and such treatment may necessarily further suppress endogenous gonadal recovery. Although phosphodiesterase-5 inhibitors do not promote recovery of the HPG axis, they may provide symptomatic relief of erectile dysfunction while endogenous gonadal recovery is awaited (53).

In summary, it is not recommended that clinicians prescribe hCG, AIs or SERMs, whether alone or in combination with androgens, to men with androgen-associated central hypogonadism as part of normal clinical care. Although testosterone treatment could be considered, subject to safety monitoring of haemoglobin and haematocrit within the reference range and serum testosterone levels targeted to the mid-normal range, this may not result in improved patient symptoms and is likely to prolong suppression of the endogenous HPG axis.

## Interventions in men seeking fertility

In selected men seeking fertility, sperm induction therapy may be considered following careful multidisciplinary discussion. Such interventions should be limited to individuals who have demonstrated sustained engagement with androgen cessation, are within a clear fertility window and in whom the potential benefits are judged to outweigh uncertain risks, considering also the partner’s age and reproductive potential. Fertility assessment should adopt a couple-centred approach, allowing discussion of reproductive goals, expected recovery timelines, female reproductive factors (including chronological age) and the uncertainties surrounding pharmacological intervention. Indeed, engagement with partners may provide valuable support and improve adherence to androgen cessation. However, in the absence of high-quality evidence addressing the safety, timing and long-term outcomes, it is premature to issue prescriptive guidance. Concerns remain regarding the potential for pharmacological sperm induction to interfere with the natural recovery timeline of the HPG axis. Where fertility treatment is considered, it should be undertaken in centres with specific expertise in sperm induction therapies and the management of men with a history of non-medical androgen use.

## Psychological interventions

Non-medical androgen use is frequently associated with numerous psychological manifestations. A history of psychological trauma, including childhood adversity, is a recognised risk factor for future non-medical androgen use (12). Men with body dysmorphic disorder, particularly muscle dysmorphia, have a substantially increased risk of future non-medical androgen use (10).

There is currently a paucity of evidence supporting psychological interventions for the prevention of non-medical androgen use or for supporting cessation. Studies examining school- or classroom-based prevention programmes in adolescents have produced mixed results (54, 55). However, small studies exploring cognitive behavioural therapy (CBT) have reported promising reductions in androgen use and improvements in psychological outcomes (56, 57). These findings require replication in larger cohorts and evaluation within a National Health Service setting to determine feasibility, acceptability and long-term effectiveness. Nevertheless, referral for formal psychological therapy may be appropriate for individuals with persistent mood symptoms, dependence or difficulties maintaining androgen cessation.

Harm-reduction services, including needle and syringe programmes (NSPs) and specialist IPED clinics, play an important role in supporting individuals who abuse androgens. Increasingly, individuals using IPEDs represent a large proportion of NSP users. In some regions of the north of England, more than 70% of individuals attending NSPs report IPED use (58). These services focus on harm reduction and risk mitigation by providing education on safer injection practices, infection prevention and broader substance misuse support. They can also serve as an important bridge to wider medical and psychological care; however, they currently do not provide a clear pathway to abstinence. Moreover, there is considerable heterogeneity in the availability and implementation of these services across the United Kingdom, despite Public Health England recommending the establishment of specialist IPED clinics to improve engagement with healthcare for this population (58, 59). Recent evidence from the Netherlands suggests that structured, clinician-led harm reduction interventions incorporating counselling, medical assessment and personalised feedback can substantially reduce androgen use. However, further studies are required to determine whether these interventions translate into sustained reductions in morbidity and facilitate long-term cessation (60).

## Future directions

Clinicians should acknowledge that the uncertainties in managing men with a history of non-medical androgen use largely reflect limitations in the current evidence base rather than a dismissal of patients’ symptoms or concerns. As new evidence emerges, approaches to assessment and management may evolve.

Further research is required to better define the natural history of non-medical androgen use withdrawal, identify predictors of biochemical and symptomatic recovery and evaluate the role of pharmacological and psychological interventions in supporting cessation and recovery. Studies should also investigate why some individuals experience persistent hypogonadal symptoms despite biochemical recovery, including potential biological mechanisms, such as altered androgen receptor signalling, alongside psychological and behavioural factors. Additional studies are also needed to determine which biomarkers are most useful for detecting undisclosed androgen use in clinical practice, improving diagnostic accuracy, monitoring recovery following androgen cessation and predicting long-term outcomes. Addressing these knowledge gaps will be essential to inform future clinical guidance, improve patient counselling and provide evidence-based care for individuals affected by non-medical androgen use.

## Declaration of interest

BG and RQ received speakers honoraria from Besins Healthcare; RQ received a speakers honorarium from Androlabs; CJ received an investigator-led grant from Logixx Pharma Ltd. RQ is a Senior Editor of *Endocrine Connections*. RQ was not involved in the peer review process of this manuscript, on which he is listed as an author.

## Funding

The Section of Endocrinology and Investigative Medicine is funded by grants from the MRC and NIHR and is supported by the NIHR Biomedical Research Centre Funding Scheme and the NIHR/Imperial Clinical Research Facility. The views expressed are those of the authors and not necessarily those of the NHS, the NIHR or the Department of Health.

## Ethics

Ethical approval was not required for this position statement as it did not involve human participants, human biological materials or the collection or use of identifiable personal data. Therefore, it did not fall within the scope of research requiring review and approval by a research ethics committee or institutional review board.
